# Therapeutic effects of composite probiotics derived from fermented camel milk on metabolic dysregulation and intestinal barrier integrity in type 2 diabetes rats

**DOI:** 10.3389/fphar.2024.1520158

**Published:** 2025-01-07

**Authors:** Tabusi Manaer, Jialehasibieke Sailike, Xin Sun, Baheban Yeerjiang, Xinhua Nabi

**Affiliations:** ^1^ School of Pharmacy, Xinjiang Medical University, Urumchi, China; ^2^ Xinjiang Key Laboratory of Biopharmaceuticals and Medical Devices, Urumchi, China; ^3^ Xinjiang Key Laboratory of Natural Medicines Active Components and Drug Release Technology, Urumchi, China; ^4^ Engineering Research Center of Xinjiang and Central Asian Medicine Resources, Ministry of Education, Urumchi, China; ^5^ Affiliated Tumor Hospital, Xinjiang Medical University, Urumchi, China; ^6^ Srational for Drug Control and Medical Device Varification of Xinjiang Military Command, Urumchi, China

**Keywords:** colon health, composite probiotics, fermented camel milk, lactic acid bacteria, tight junction proteins, type 2 diabetes, yeast

## Abstract

**Background:**

In the Kazakh community of Xinjiang, China, fermented camel milk has been traditionally used to manage diabetes. This study evaluates the effects of composite probiotics derived from fermented camel milk (CPCM) on metabolic disturbances in a rat model of Type 2 diabetes (T2DM).

**Methods:**

T2DM was induced in Wistar rats using streptozotocin. Experimental groups included a diabetic control, Metformin, and low- and high-dose CPCM. Measurements over 6 weeks included body weight (BW), fasting blood glucose (FBG), oral glucose tolerance test (OGTT), glycated hemoglobin (HbA1c), C-peptide (CP), lipid profiles, inflammatory markers, fecal short-chain fatty acids (SCFAs), and tight junction protein expression in colonic tissues.

**Results:**

High-dose CPCM significantly increased BW by 22.2% (*p* < 0.05) and reduced FBG by 6.5 mmol/L (*p* < 0.001). The OGTT AUC decreased by 40.1% (*p* < 0.001), and HbA1c levels fell by 22.9% (*p* < 0.01). CP levels rose by 21.8% (*p* < 0.05). Lipid profiles improved: TC decreased by 40.0%, TG by 17.1%, and LDL-C by 30.4% (all *p* < 0.001). Fecal SCFAs, including acetate (75.4%, *p* < 0.001), methyl acetate (18.9%, *p* < 0.05), and butyrate (289.9%, *p* < 0.001), increased, with total SCFAs rising by 89.7% (*p* < 0.001). Inflammatory markers IL-1β (12.7%, *p* < 0.01), TNF-α (16.7%, *p* < 0.05), and IL-6 (17.3%, *p* < 0.01) were significantly reduced. Tight junction protein expression (ZO-1, occludin, claudin-1) and mucin (MUC2) in colonic tissues increased (*p* < 0.05). CPCM treatment also reduced serum total bile acids by 24.9%, while hepatic and fecal bile acids increased by 114.0% and 37.8% (all *p* < 0.001). CPCM lowered serum DAO, D-lactate, and LPS levels (all *p* < 0.001). mRNA levels of TGR5 and CYP7A1 in the liver, and TGR5 and FXR in the colon, were markedly elevated (all *p* < 0.001). Histological examinations revealed reduced pancreatic inflammation and hepatic steatosis, with restored colonic structure.

**Conclusion:**

CPCM treatment significantly improved metabolic dysregulation in the T2DM rat model, reducing blood glucose and lipid levels, enhancing intestinal barrier function, and increasing insulin secretion. These findings highlight the therapeutic potential of CPCM in T2DM management and probiotics’ role in metabolic health.

## 1 Introduction

Type 2 diabetes mellitus (T2DM) is a prevalent metabolic disorder characterized by insulin resistance, hyperglycemia, and dyslipidemia, presenting significant health challenges globally (NCD Risk Factor Collaboration, 2024; Strati et al., 2024; Ogurtsova et al., 2022). The management of T2DM often involves lifestyle modifications and pharmacological interventions, yet these approaches can fall short in addressing the underlying metabolic dysregulation and associated complications (Adler et al., 2024; El-Nashar et al., 2024a; El-Nashar et al., 2024b; ElSayed et al., 2024). Recent research has begun to explore the role of dietary components, particularly fermented foods, in improving metabolic health (Leeuwendaal et al., 2022; Zhang et al., 2024).

Research indicates that Kazakhs exhibit a relatively high obesity rate while simultaneously having a low incidence of diabetes (Li et al., 2015; Shen et al., 2024; Yan et al., 2015). This raises the question of whether this phenomenon is related to their long-term consumption of traditional fermented products. Fermented camel milk has been traditionally utilized in various cultures for its health benefits, particularly in the management of diabetes among the Kazakh ethnic group (Manaer et al., 2015). This milk contains higher levels of insulin-like proteins, unique fatty acids, and bioactive peptides, which may contribute to its antidiabetic properties (Bilal et al., 2024a; Bilal et al., 2024b). Additionally, the presence of probiotics offers further advantages by modulating gut microbiota, enhancing intestinal barrier function, and reducing inflammation—factors that are critical in the pathophysiology of T2DM (Kamińska et al., 2022; Zhai et al., 2021).

In our previous study, we isolated four yeast species and ten strains of lactic acid bacteria from traditionally fermented camel milk sourced from the Xinjiang region in China. These isolates were characterized by the Institute of Microbiology, Chinese Academy of Agricultural Sciences. Our preliminary research has identified that the fourteen composite probiotics used in our study have the potential to improve diabetes prognosis (Wang et al., 2020a; Wang et al., 2020b). According to the International Scientific Association for Probiotics and Prebiotics (ISAPP), probiotics in food or supplements can be associated with specific health claims (Hill et al., 2014). Therefore, the fourteen probiotics we are using fall within the category of probiotics. These findings underscore the potential of these probiotic strains to contribute positively to metabolic health and diabetes management.

Recent studies have indicated that the gut microbiome plays a pivotal role in metabolic regulation (Wu et al., 2021). Dysbiosis, or the imbalance of gut bacteria, has been linked to insulin resistance and chronic inflammation, common features in T2DM (Lee et al., 2020). Probiotics, particularly those derived from fermented foods, can restore microbial balance, promote the production of short-chain fatty acids (SCFAs), and enhance gut health (Fusco et al., 2023; Mazhar et al., 2023). These mechanisms are particularly relevant in the context of T2DM, where improving gut integrity and reducing systemic inflammation can lead to better metabolic outcomes (Palmnäs-Bédard et al., 2022).

Composite probiotics from fermented camel milk (CPCM) represent a novel approach to harnessing these benefits (Wang et al., 2020a; Wang et al., 2020b). The synergistic effects of lactic acid bacteria and other microorganisms present in CPCM may improve metabolic dysregulation by promoting insulin sensitivity, enhancing lipid profiles, and reinforcing intestinal barrier integrity (Manaer et al., 2021). Moreover, the unique properties of camel milk, such as its anti-inflammatory and antioxidant effects, may further augment the therapeutic potential of CPCM in managing T2DM (Nabi et al., 2016; Wang et al., 2023).

This study aims to investigate the impact of CPCM on metabolic dysregulation and intestinal barrier function in a T2DM rat model. By elucidating the specific mechanisms through which CPCM exerts its effects, we hope to provide insights into its potential as a dietary intervention for T2DM. The findings will not only contribute to the understanding of the health benefits of fermented camel milk but also highlight the importance of probiotics in the management of metabolic disorders.

The unique composition of camel milk, combined with the beneficial effects of its fermented probiotics, positions CPCM as a promising candidate for addressing the complexities of T2DM. By focusing on both metabolic improvements and gut health, this research aims to underscore the therapeutic potential of CPCM as an integrative approach to diabetes management.

## 2 Materials and methods

### 2.1 Probiotic sources

In our previous study, we isolated four yeast species and ten strains of lactic acid bacteria from traditionally fermented camel milk sourced from the Xinjiang region in China. These isolates were characterized by the Institute of Microbiology, Chinese Academy of Agricultural Sciences. Based on the current taxonomy of lactic acid bacteria (LAB) (see: http://lactobacillus.ualberta.ca/), the identified LAB included strains such as *Lactobacillus pentosus*, *Limosilactobacillus mucosae*, *Lactobacillus helveticus*, *Lactobacillus paracasei subsp. tolerans*, and *Lactiplantibacillus plantarum subsp plantarum*, among others. The yeast species included *Issatchenkia orientalis* and *Kluyveromyces marxianus*, among others.

### 2.2 Reagents and instruments

De Man, Rogosa, and Sharpe (MRS) Broth, Malt Juice Broth, and MRS Solid Culture Media—Obtained from Qingdao Rishui Biotechnology Co., Ltd. Sha’s Agar Medium—Acquired from Hangzhou Microbial Reagent Co., Ltd. Metformin—Sourced from Shanghai Squibb Pharmaceutical Co., Ltd. Polyformaldehyde—Obtained from Beijing Solarbio Company. Sodium Pentobarbital—Purchased from Merck & Co., Germany (Batch No. 202007). ELISA Kits for Rat glycated hemoglobin (HbA1c), C-peptide (CP), interleukin-1 beta (IL-1β), tumor necrosis factor-alpha (TNF-α), interleukin-6 (IL-6), and total bile acid - Procured from Wuhan Huamei Biological Co., Ltd. Rat Lipopolysaccharides (LPS) (JYM0937Ra), Diamine Oxidase (DAO) (JYM0013Ra), and D-Lactic Acid (D-Lactate) (JYM0693Ra) ELISA Kits—Sourced from Wuhan Gene Beauty Technology Co., Ltd. Anhydrous Ethanol - Purchased from Tianjin Beilian Fine Chemicals Development Co., Ltd. DNA Marker and 2× PCR Master Mix—Acquired from Beijing Tiangen Biochemical Technology Co., Ltd. 10× Loading Buffer and DNase/RNase-free Deionized Water—Obtained from SolarBio, Beijing, China. 5× TBE Buffer - Sourced from Shanghai Shenggong Biotechnology Co., Ltd. Primers—Supplied by Biotechnology (Shanghai) Co., Ltd. Antibodies—Obtained from Affiniti Company. DEPC-treated Water—Purchased from SolarBio, Beijing, China (Catalog No. BL510A). Trizol Reagent—Purchased from Thermo Fisher Scientific, United States (Catalog No. 15596026). cDNA Reverse Transcription Kit—Purchased from Thermo Fisher Scientific, United States (Catalog No. K1728). SYBR Green PCR Kit—Purchased from Takara, Japan (Catalog No. AJ12465A). RIPA Lysis Buffer—Purchased from SolarBio, Beijing, China (Catalog No. R0020). BCA Protein Assay Kit—Purchased from Thermo Fisher Scientific, United States (Catalog No. 1862633). Goat Anti-Rabbit Secondary Antibody—Purchased from Abcam, United States (Catalog No. ab8227).

The XQ-LS-50 vertical pressure steam sterilizer was procured from Shanghai Boxun Company, while the AC-S clean bench was acquired from Suzhou Antai Air Technology Co., Ltd. Eppendorf Company provided the pipettes. The DNP-9082 electric constant temperature incubator was sourced from Shanghai Jinghong Experimental Equipment Co., Ltd. The AY120 electronic analytical balance was supplied by Shimadzu (Japan). The LXJ-2B low-speed, large-capacity centrifuge was obtained from Shanghai Anting Scientific Instrument Factory. Blood glucose meters and their corresponding test strips were acquired from Roche Diagnostic Products Shanghai Co., Ltd. The DHG-9070A electric blast drying oven was sourced from Shanghai Yiheng Scientific Instruments Co., Ltd. The CA-2000CDA fully automated enzyme labeling instrument was obtained from Bio-Rad (United States). The SCOTSMRN AF 100 ice maker was sourced from STOSMRN Company. The electrophoresis apparatus was also acquired from Bio-Rad (United States). The MLS-3750 high-pressure steam sterilizer was supplied by SANYO Company. The DanoDrop 1000 nucleic acid and protein detector, real-time fluorescence quantitative PCR instrument, and C1000 PCR amplification instrument were all sourced from Bio-Rad (United States). Lastly, the IQ5 electric constant temperature water bath was procured from Shanghai Jinghong Experimental Equipment Co., Ltd. The Agilent 7890A Gas Chromatograph (Agilent Technologies, Santa Clara, CA, United States) was equipped with a flame ionization detector (FID) and a DB-WAX capillary column (30 m × 0.25 mm, 0.25 µm film thickness).

### 2.3 Preparation of composite probiotics derived from fermented camel milk (CPCM)

#### 2.3.1 Activation of probiotic strains

The freeze-dried formulations of 14 distinct strains of lactic acid bacteria and yeast used in this investigation were rehydrated, encapsulated, and inoculated. These strains were subsequently cultured at 37°C to generate the first generation. The cultures underwent further expansion in De Man, Rogosa, and Sharpe (MRS) broth for an additional 2–3 generations. After this incubation period, the cultures were harvested and stored at 4°C for prospective applications. Comprehensive labeling and meticulous documentation of the preserved cultures were implemented to ensure traceability.

#### 2.3.2 Assessment of probiotic viability

The viability of ten LAB strains was evaluated utilizing the Azurol method, while four yeast species were assessed through methylene blue reduction tests. Azurol method is a colorimetric technique allowing for the determination of the metabolic activity of the bacteria. In this method, the LAB strains were cultured in selective media under anaerobic conditions to promote optimal growth. After incubation, a specific volume of the culture was mixed with Azurol dye, which changes color in response to the metabolic activity of the bacteria. The intensity of the color change is proportional to the number of viable cells present in the sample. This method provides a reliable measure of the viability of LAB based on their ability to reduce the Azurol dye, indicating active metabolism and growth. For the assessment of four yeast species, we utilized methylene blue reduction tests. In this procedure, yeast cultures were incubated in a medium containing methylene blue, which acts as an indicator of cellular respiration. Viable yeast cells can reduce methylene blue to a colorless form, while non-viable cells retain the blue color. The process involves adding a defined concentration of methylene blue to the yeast culture and incubating it for a specific period. The reduction of the dye is then visually assessed or measured spectrophotometrically. This method is effective for determining the viability of yeast cells, as it correlates with their metabolic activity and overall health. Both assessment methods are crucial for ensuring that the probiotic strains tested in this study are viable and capable of exerting their beneficial effects in the context of metabolic dysregulation and intestinal health.

#### 2.3.3 Quantification and concentration adjustment of live bacterial counts

To ascertain the viable counts of lactic acid bacteria, samples were serially diluted from 10 ^-1 to 10 ^-10, plated onto MRS agar, incubated at 37°C for 48 h, and subsequently enumerated. For the viability assessment of yeast, samples were diluted from 10 ^-1 to 10 ^-8, cultured on Sabouraud agar, and incubated at 30°C for 48 h prior to counting the yeast colonies for accurate quantification.

#### 2.3.4 Preparation of skimmed camel milk

Fresh camel milk was procured from a reliable supplier and subjected to defatting via high-speed centrifugation at 4,000 rpm for 15 min. The lipid layer was eliminated, and the defatted milk was homogenized. The resulting defatted camel milk was then sterilized at 95°C for approximately 20 min before being cooled to room temperature.

#### 2.3.5 Preparation of CPCM with defatted sterilized camel milk

In accordance with the established formulation criteria for CPCM, both lactic acid bacteria and yeast were proportionately integrated with the defatted sterilized camel milk to formulate the composite probiotic reagent. The final product was then preserved at −80°C for subsequent use.

### 2.4 Animals and treatments

Fifty male Wistar rats, 6 weeks old and weighing 180 ± 20 g, were obtained from the Experimental Animal Center of Xinjiang Medical University and were housed in the Barrier Environment Animal Laboratory at the same institution (Production and Use License: SCXK (XIN)2018-0002). The study received approval from the Institutional Animal Care and Use Committee (IACUC) of Xinjiang Medical University, under ethical review approval identifier IACUC-20220312-10. The housing environment conformed to specific pathogen-free (SPF) standards, maintaining a controlled light cycle, a temperature of 23°C ± 1°C, and a relative humidity of approximately 50% ± 5%.

Following an adaptation period of 3 days, the rats were divided into two groups. Eight rats were randomly assigned to the normal control group and were provided with standard rat chow, while the remaining forty-two rats were classified as model rats and were fed a high-sugar, high-fat diet sourced from the Experimental Animal Center of Xinjiang Medical University. All rats had unrestricted access to water. This feeding regimen continued for 8 weeks, during which the body weight (BW) of the rats was recorded weekly. At the conclusion of the 8-week period, the rats underwent a fasting period of 12 h while still having access to water. The normal control group received an intraperitoneal injection of a trisodium citrate buffer solution (0.1 mol/L, pH 4.4). In contrast, the model group was administered an intraperitoneal injection of streptozotocin (STZ) at a dose of 35 mg/kg, with the STZ dissolved in the same trisodium citrate buffer solution. Blood samples were collected from the tail tip 3 days post-injection, following a 12-h fasting period, to assess fasting blood glucose (FBG) levels using a calibrated blood glucose meter. A rat was classified as diabetic if its FBG levels were ≥11.1 mmol/L. To validate the stability and success of the diabetes model, FBG measurements were taken at one and 2 weeks following the STZ injection, again following a 12-h fasting period. A minimum of three FBG readings ≥11.1 mmol/L were necessary to confirm the establishment of the diabetes model. Ultimately, thirty-two rats were successfully induced to develop type 2 diabetes for subsequent experimental investigations.

Preparation of Trisodium Citrate Buffer Solution: The trisodium citrate buffer solution (0.1 mol/L, pH 4.4) was prepared as follows: Component A was created by dissolving 2.94 g of trisodium citrate in 100 mL of distilled water to achieve a 0.1 mol/L concentration. Component B was prepared by dissolving 2.10 g of citric acid in 100 mL of distilled water to form a 0.1 mol/L solution. The working solution (pH 4.4) was then obtained by mixing components A and B in a volume ratio of 1:1.32.

Preparation of STZ Solution: For each injection, 0.2 g of STZ was meticulously weighed and dissolved in 20 mL of the 0.1 mol/L working solution, resulting in a final concentration of 1%. It is imperative that the STZ solution be prepared under ice bath conditions immediately prior to use and administered promptly in a dark environment to ensure its stability.

A total of eight SPF male Wistar rats were designated to the normal control group (NC) and were administered sterilized camel milk via gavage at a dose of 10 mL/kg. Thirty-two diabetic model rats were randomly allocated to four treatment cohorts: the diabetes model group (DM), which received sterilized camel milk at a dosage of 10 mL/kg; the Metformin group (Metformin), which was treated with Metformin at a concentration of 300 mg/kg in a volume of 10 mL/kg; and two CPCM treatment groups classified as low-dose (Low dose) and high-dose (High dose). The low-dose group received 1.0 × 10 ^ 8 CFU/mL of lactic acid bacteria and 1.0 × 10 ^ 6 CFU/mL of yeasts, while the high-dose group was given a solution containing 1.0 × 10 ^ 10 CFU/mL of lactic acid bacteria and 1.0 × 10 ^ 8 CFU/mL of yeasts (Manaer et al., 2021; Wang et al., 2020b). dissolved in sterilized camel milk at the same dosage of 10 mL/kg. All groups underwent a 6-week gavage treatment. In the final week, fecal samples were collected and stored at −80°C for further analysis. Prior to euthanasia, all rats were subjected to a 12-h fasting period, during which they had no access to food or water. Anesthesia was induced through intraperitoneal injection of 1% sodium pentobarbital at a dose of 45 mg/kg of body weight (BW).

### 2.5 Testing indicators

#### 2.5.1 General symptom monitoring

The fur color and activity levels of the diabetic rats were assessed three times weekly. Daily evaluations included monitoring water intake, food remnants, and bedding moisture, with timely cage cleaning and bedding replacement as needed.

#### 2.5.2 Body weight and fasting blood glucose measurement

Post-modeling, BW and FBG levels were recorded weekly at standardized intervals under fasting conditions (note: water was not withheld).

#### 2.5.3 Oral glucose tolerance test

At the study’s conclusion, all rats underwent a 12-h fasting period, followed by gavage administration of a glucose solution at a dosage of 2 g/kg based on average BW. Blood samples were collected from the tail tips at specific time points: 0, 30, 60, 90, and 120 min, to measure blood glucose levels during the OGTT. The resulting data were utilized to create a blood glucose response curve, and the area under the curve (AUC) was calculated to evaluate the glucose tolerance of each group based on the AUC magnitude.

#### 2.5.4 Collection of blood and organ tissues

Following anesthesia, blood samples were drawn from the abdominal aorta, and euthanasia was performed via cervical dislocation. Serum was subsequently obtained by centrifugation at 3,500 rpm for 15 min at 4°C for additional analysis. Subsequent to blood collection, the rats were dissected to procure tissues. Pancreatic, hepatic, colonic, and other organ samples were swiftly transferred to liquid nitrogen for storage at −80°C. Specimens designated for pathological assessment were preserved in a 4% formaldehyde solution.

#### 2.5.5 Biomarker quantification

The levels of serum biomarkers, including total cholesterol (TC), triglycerides (TG), low-density lipoprotein cholesterol (LDL-C), and high-density lipoprotein cholesterol (HDL-C), were measured utilizing a fully automated biochemical analyzer. The concentrations of glycated hemoglobin (HbA1c), C-peptide (CP), diamine oxidase (DAO), D-lactic acid (D-Lactate), lipopolysaccharides (LPS), interleukin-1 beta (IL-1β), tumor necrosis factor-alpha (TNF-α), and interleukin-6 (IL-6) and serum, hepatic, and fecal bile acids were evaluated using enzyme-linked immunosorbent assay (ELISA) kits, adhering to the protocols outlined by the manufacturer.

#### 2.5.6 Total RNA extraction from colon and liver tissues

Pre-chill a 1 mL tube of TRIzol Reagent on ice. Weigh 50–100 mg of tissue and place it in a mortar. Add liquid nitrogen and grind the tissue thoroughly until it becomes a fine powder. Quickly transfer the powdered tissue into the tube containing TRIzol Reagent and mix thoroughly. Centrifuge at 12,000 rpm for 10 min in a 4°C centrifuge and collect the supernatant. Add 200 μL of chloroform to the supernatant, vigorously shake the tube up and down for 15 s to ensure proper mixing, and let it sit at room temperature for 3 min. Centrifuge again at 12,000 rpm for 10 min at 4°C and collect the supernatant. Add 500 μL of isopropanol to the supernatant and let it sit for 10 min, then centrifuge at 12,000 rpm for 10 min at 4°C. (Note: The white precipitate formed is RNA.) Wash the precipitate by adding 1 mL of 75% ethanol, centrifuge at 7,500 rpm for 5 min at 4°C, and repeat this wash twice. After removing the supernatant completely, place the tube in a clean bench and air dry for 3 min until no liquid remains on the white precipitate. Add 40 μL of DEPC water to dissolve the RNA, ensuring the white precipitate is fully dissolved. Take 3 μL of the RNA solution to measure RNA concentration and purity. Adjust the RNA concentration of each sample to be consistent by diluting with the appropriate volume of DEPC water based on RNA purity, and store at −20°C for future use.

Synthesis of cDNA via Reverse Transcription. Take a sterilized PCR tube and add 2 μg of RNA solution. Then, add 1 μL of oligo (dT) primer and bring the total volume to 12 μL with DEPC water. Place the PCR tube in a PCR machine and incubate at 65°C for 5 min. Add the appropriate amounts of Buffer, 10 mM dNTP Mix, Ribolock RNAse inhibitor, and reverse transcriptase to the tube, and mix thoroughly. Incubate the tube in the PCR machine at 42°C for 1 h, followed by 70°C for 5 min to inactivate the reverse transcriptase.

RT-PCR: Take sterilized 200 μL PCR tubes and prepare the reaction mixture, creating three tubes for each reverse transcription product sample. Perform PCR amplification. Include the RT-PCR primer sequences. Table1 shows the RT-PCR primer sequence. Analyze results using β-actin as an internal control. The expression level in the NC group is set to 1, and expression fold change is calculated using the formula 2 ^ (-ΔΔCt).

**TABLE 1:** Shows the RT-PCR primer sequence.

Primer	Sequence (5′to3′)	Temperature (°C)
TGR5	F:GAGCCATCAGGGGTACTGGT	60
	R:GGCTGCAACACTGCCATGTA	
FXR	F:CGTCGGAAGTGCCAGGATTGC	60
	R:CCTTCGCTGTCCTCATTCACTGTC	
CYP7A1	F:AAAGCTGGCTGAGGGATTGA	61
	R:GCCCAGAGAATAGCGAGGTG	
CYP21A1	F:ACCACGGCTACCACGCTCTC	62
	R:AACGCAGCACCTCGGCAATG	
β-actin	F:CTGAACCCTAAGGCCAACCG	60
	R:GACCAGAGGCATACAGGGACAA	

#### 2.5.7 Determination of short-chain fatty acids in feces by gas chromatography

##### 2.5.7.1 Preparation of standard stock solution

A mixed standard stock solution containing acetic acid, propionic acid, and butyric acid at concentrations of 200 mmol/L, 100 mmol/L, and 100 mmol/L, respectively, was prepared using standard short-chain fatty acids. This solution was stored at −20°C for future use.

##### 2.5.7.2 Fecal sample processing

Two hundred milligrams of rat fecal samples were weighed into a sterile centrifuge tube. To this, 1 mL of sterile water was added, along with 2-ethylbutyric acid as the internal standard, achieving a final concentration of 2 mmol/L. The pH of the mixture was adjusted to approximately 2–3 using sulfuric acid. The sample was vortexed for 3 min to ensure thorough mixing, followed by centrifugation at 12,000 rpm for 10 min. The supernatant was carefully collected and mixed with 1 mL of diethyl ether for extraction. After centrifugation at 12,000 rpm for 15 min, the supernatant was filtered through a 0.45 μm organic membrane filter before being analyzed.

##### 2.5.7.3 Gas chromatography conditions

(1) A DB-WAX capillary column (30 m × 0.25 mm, 0.25 µm film thickness) was used, with nitrogen as the carrier gas. The injection volume was 1 μL, and the flow rate was set at 1 mL/min. (2) Hydrogen flow rate: 40 mL/min; air flow rate: 400 mL/min; tail gas flow rate: 40 mL/min. (3) Injection port temperature: 220°C. (4) Temperature program: The initial column temperature was set at 110°C for a hold time of 2.5 min, followed by an increase to 150°C at a rate of 8°C/min, with a hold time of 8 min (5) Detector temperature: 230°C. (6) Total run time: 15.5 min.

#### 2.5.8 Pathological evaluation of pancreatic, hepatic and colonic tissues

Hematoxylin and eosin (HE) staining was employed to examine the morphological characteristics of pancreatic, hepatic and colonic tissues from rats. The expression of tight junction proteins and mucin genes within colonic tissue is essential for preserving the integrity of the intestinal barrier, which serves to protect against pathogens and toxins. Dysregulation of these genes has been implicated in various gastrointestinal disorders such as inflammatory bowel disease and colorectal cancer. Investigating the regulatory mechanisms behind the expression of tight junction proteins and mucin genes in colonic tissue could yield valuable insights into the pathophysiology of these conditions and reveal potential therapeutic strategies. The expression levels of tight junction protein genes—specifically ZO-1, Occludin, and Claudin-1—along with the mucin protein MUC2, were assessed in colonic tissue. Both tight junction and mucin proteins are pivotal in maintaining the intestinal barrier’s integrity and providing defense against harmful substances. Tight junction proteins create a selective barrier between epithelial cells, regulating the passage of molecules and ions, while mucin proteins contribute to the formation of a protective mucus layer over the epithelial surface. A thorough understanding of the regulatory pathways governing these proteins in colonic tissue can enhance our knowledge of gastrointestinal health and disease. Immunohistochemistry (IHC) techniques were utilized to determine the expression levels of tight junction proteins ZO-1, Occludin, Claudin-1, and the mucin protein MUC2 in colonic samples.

### 2.6 Statistical analysis

Statistical analyses were performed using SPSS version 23.0. Data are presented as mean ± standard deviation (mean ± SD), and intergroup comparisons were conducted using Student’s t-test to evaluate the statistical significance (set at *p < 0.05, **p < 0.01, ***p < 0.001) between two given experimental groups: pairwise comparison of each sample. For comparisons involving more than two groups, one-way analysis of variance (ANOVA) followed by Tukey’s *post hoc* test was employed. All pairwise comparisons were made to ensure the reliability of the results.

## 3 Results

### 3.1 Overview of rat condition

The NC group rats were observed to display increased levels of activity, a vibrant and glossy fur, and normal feeding behaviors. In contrast, the T2DM rats exhibited diminished activity, signs of mental lethargy, a significant increase in water intake, polyuria, and a dull coat. After several weeks of intervention, there was a partial improvement in the aforementioned pathological symptoms in both the Metformin and High dose groups.

### 3.2 Impact of CPCM on body weight in T2DM rats

Before the administration of STZ, the BW of the T2DM rats on a high-fat diet was significantly greater than that of the NC group, with a statistically significant difference (*p* < 0.05). The rats were subsequently assigned to various treatment groups, with no significant differences in BW observed among the groups (*p* > 0.05). Throughout the intervention period, T2DM rats continued to experience BW loss, with a notable decrease beginning in the fourth week. When compared to the NC group, all T2DM groups demonstrated significant weight loss (*p* < 0.01). By the sixth week, significant BW gain differences were noted in both the Metformin (387.87 ± 36.88 g) and High dose (386.88 ± 35.33 g) groups relative to the DM (316.58 ± 29.52 g) group (*p* < 0.05), indicating the efficacy of CPCM in alleviating BW loss in T2DM rats. These findings are summarized in Table 2.

**TABLE 2:** Impact of CPCM on Body Weight (BW) in T2DM Rats The study included a sample size of 8 rats, with results expressed as mean ± standard deviation (SD).

Group	Before treatment (g)	Week 1 (g)	Week 2 (g)	Week 3 (g)	Week 4 (g)	Week 5 (g)	Week 6 (g)
NC	393.07 ± 23.68	404.65 ± 24.50	416.27 ± 30.08	426.87 ± 27.52	439.65 ± 23.84	446.72 ± 24.92	454.10 ± 22.77
DM	428.75 ± 19.19**	403.35 ± 23.18	379.70 ± 25.19	359.85 ± 28.87	341.30 ± 31.30***	329.93 ± 29.51***	316.58 ± 29.52***
Metformin	430.48 ± 33.54**	408.80 ± 29.94	387.97 ± 30.59	376.37 ± 33.17	381.50 ± 35.44***	385.20 ± 35.35	387.87 ± 36.88^#^
Low dose	427.22 ± 24.72**	401.15 ± 27.41	374.13 ± 23.79	361.07 ± 24.02	360.40 ± 28.23***	361.88 ± 27.64	364.00 ± 27.55
High dose	426.47 ± 18.70**	402.20 ± 22.53	381.45 ± 23.62	374.67 ± 31.79	379.17 ± 34.98***	383.17 ± 34.03	386.88 ± 35.33^#^

**p < 0.05, ***p < 0.01 vs. NC, ^#^ *p* < 0.05 vs. DM. These statistical comparisons highlight the significant differences between the groups in the study.

### 3.3 Impact of CPCM on glucose metabolism in T2DM rats

#### 3.3.1 Impact of CPCM on fasting blood glucose levels in T2DM rats

Initial data indicated that FBG levels in the DM group were significantly elevated compared to the NC group (*p* < 0.001). No significant differences in FBG levels were observed among the experimental groups relative to the DM group (*p* > 0.05), confirming the reliability of the T2DM model in the context of the randomized and rational experimental design.

Following oral administration of CPCM, FBG levels in the DM group rats remained consistently elevated over a 6-week period, with significant differences compared to the NC group (*p* < 0.001). In the Metformin group, FBG levels began to decline from the second week, achieving significant differences from the DM group by the third week (*p* < 0.05), with heightened significance from the fifth week onward (*p* < 0.001).

The Low dose group exhibited a downward trend in FBG levels by the fifth week, achieving statistical significance compared to the DM group by the sixth week (*p* < 0.01). Likewise, the High dose group showed a reduction in FBG levels starting in the third week, with significant differences from the DM group evident from the fourth week (*p* < 0.05) and reaching significance by the fifth week (*p* < 0.01). By the sixth week, the difference was extremely significant (*p* < 0.001).

No significant differences in FBG reduction were observed between the High dose and Metformin groups (*p* > 0.05). The Low dose group demonstrated significant differences in FBG levels beginning in the third week, achieving statistical significance (*p* < 0.05). These results are presented in Table 3.

**TABLE 3:** Impact of CPCM on Fasting Blood Glucose (FBG) Levels in T2DM Rats The study included a sample size of 8 rats, with results expressed as mean ± standard deviation (SD).

Group	Before treatment (mmol/L)	Week 1 (mmol/L)	Week 2 (mmol/L)	Week 3 (mmol/L)	Week 4 (mmol/L)	Week 5 (mmol/L)	Week 6 (mmol/L)
NC	5.3 ± 0.4	5.2 ± 0.4	5.1 ± 0.5	5.1 ± 0.4	5.2 ± 0.4	5.1 ± 0.5	5.2 ± 0.5
DM	22.3 ± 3.0***	23.2 ± 4.2***	23.6 ± 3.4***	24.2 ± 3.8***	24.6 ± 3.2***	25.3 ± 3.0***	25.1 ± 2.6***
Metformin	22.9 ± 4.7	23.0 ± 2.8	22.5 ± 2.5	20.0 ± 2.5^#^	20.3 ± 2.1^##^	18.3 ± 3.2^###^	17.8 ± 2.5^###^
Low dose	22.5 ± 3.5	23.5 ± 3.1	23.7 ± 2.7	23.8 ± 2.0^▲^	24.1 ± 2.5^▲^	23.0 ± 3.2^▲^	21.6 ± 2.4^##▲^
High dose	22.6 ± 3.4	22.9 ± 2.5	22.4 ± 2.8	21.8 ± 2.9	21.1 ± 3.1^#^	20.2 ± 3.1^##^	18.6 ± 1.7^###^

***p < 0.001 vs. NC; ^#^ *p* < 0.05, ^##^ *p* < 0.01, ^###^ *p* < 0.001 vs. DM; ^▲^ *p* < 0.05 vs. Metformin. These statistical comparisons highlight the significant differences between the groups in the study.

#### 3.3.2 Impact of CPCM on oral glucose tolerance test and area under the curve in T2DM rats

The assessment of the OGTT and the calculation of the AUC are critical parameters for evaluating glucose metabolism. The results show that the OGTT and AUC values in the DM group were significantly higher than those in the NC group, with a marked difference (*p* < 0.001). After a 6-week treatment period, the measurements for OGTT and AUC in the Metformin, Low dose, and High dose groups demonstrated significant reductions compared to the DM group, with highly significant differences noted (*p* < 0.001). The improvement in OGTT and AUC in the Low dose group was less pronounced, suggesting a differential effect relative to the Metformin group (*p* < 0.05). The High dose group’s improvement was comparable to that of the Metformin group, with no statistically significant differences observed. The detailed results are presented in Table 4.

**TABLE 4:** Impact of CPCM on Oral Glucose Tolerance Test (OGTT) and Area Under the Curve (AUC) in T2DM Rats The study included a sample size of 8 rats, with results expressed as mean ± standard deviation (SD).

Group	0 min (mmol/L)	30 min (mmol/L)	60 min (mmol/L)	90 min (mmol/L)	120 min (mmol/L)	AUC
NC	5.43 ± 0.27	6.68 ± 0.47	6.22 ± 0.32	5.60 ± 0.18	5.20 ± 0.37	8,072.85 ± 36.35
DM	25.28 ± 2.19^***^	30.37 ± 2.28^***^	28.82 ± 1.69^***^	27.62 ± 1.41^***^	26.97 ± 1.13^***^	3,885.35 ± 610.34^***^
Metformin	17.90 ± 2.49^###^	21.85 ± 2.17^###^	20.72 ± 2.03^###^	19.83 ± 1.86^###^	18.58 ± 1.82^###^	2,138.00 ± 350.56^###^
Low dose	22.58 ± 2.71^#▲^	26.90 ± 2.26^##▲▲^	24.88 ± 1.29^###▲▲^	23.97 ± 1.69^###▲▲^	23.00 ± 1.40^####▲▲^	2,725.00 ± 331.89^###▲▲^
High dose	18.70 ± 1.66^###^	23.42 ± 1.88^###^	21.77 ± 1.98^###^	20.92 ± 1.82^###^	19.78 ± 2.12^###^	2,325.75 ± 306.25^###^

***p < 0.001 vs. NC; ^#^ *p* < 0.05, ^##^ *p* < 0.01, ^###^ *p* < 0.001 vs. DM; ^▲^ *p* < 0.01, ^▲▲^ *p* < 0.001 vs. Metformin. These statistical comparisons highlight the significant differences between the groups in the study.

#### 3.3.3 Impact of CPCM on glycosylated hemoglobin (HbA1c) and C-Peptide in T2DM rats

**FIGURE 1:**
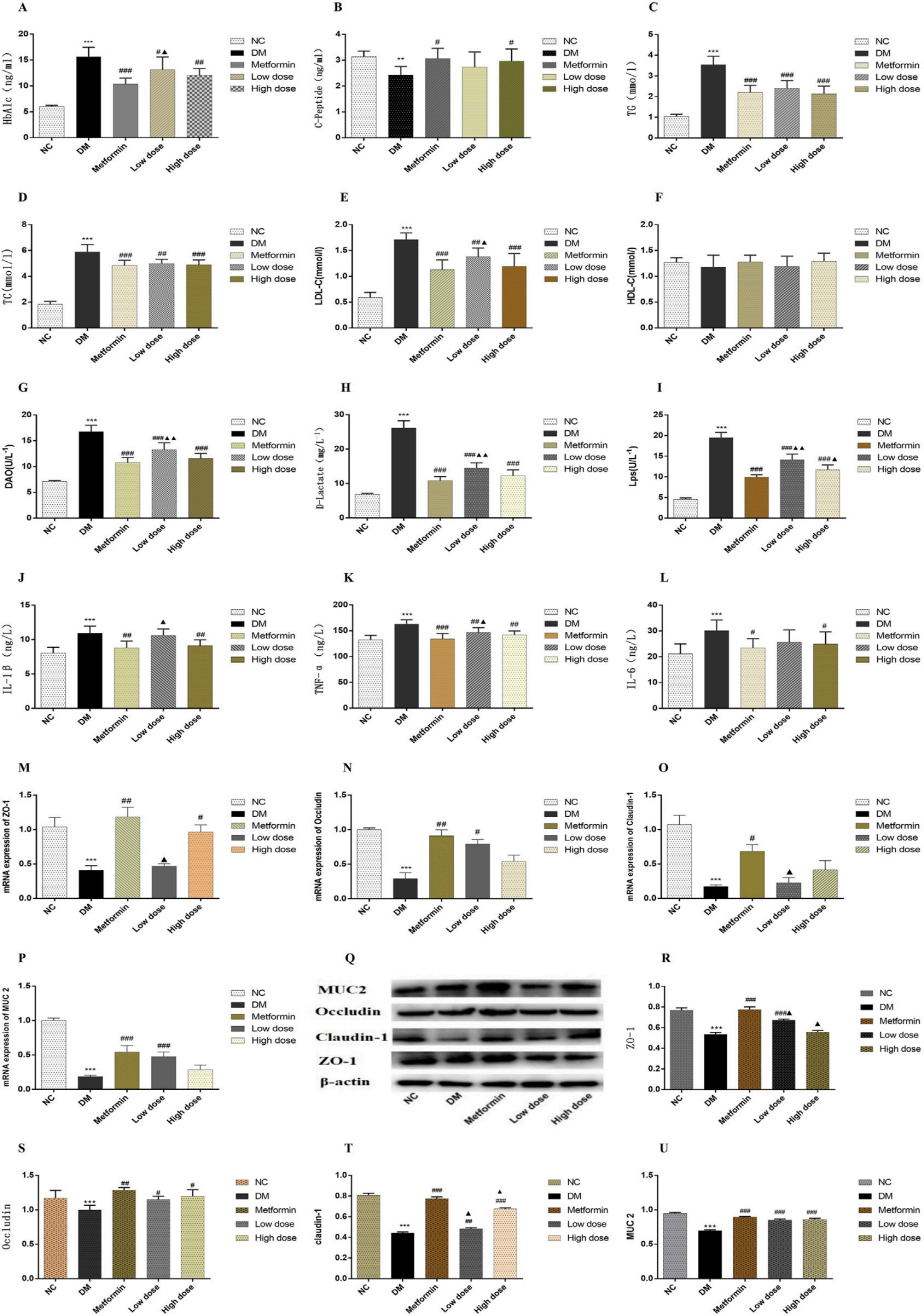
Impact of CPCM on Biomarker Levels and Protein Expression in Colon Tissues. This figure presents the effects of CPCM on serum levels of various biomarkers, including **(A)** HbA1c, **(B)** CP, **(C)** TC, **(D)** TG, **(E)** LDL-C, **(F)** HDL-C, **(G)** DAO, **(H)** D-lactate, **(I)** LPS, **(J)** IL-1β, **(K)** TNF-α, and **(L)** IL-6. Additionally, the mRNA expression levels of tight junction proteins, including **(M)** ZO-1, **(N)** Occludin, and **(O)** Claudin-1, as well as mucin **(P)** MUC2, were assessed. Furthermore, **(Q)** Western blot analyses were conducted to evaluate the protein expression levels of tight junction proteins [**(R)** ZO-1, **(S)** Occludin, **(T)** Claudin-1] and mucin [**(U)** MUC2] in colon tissues. The study included a sample size of 8 rats, with results expressed as mean ± standard deviation (SD). **p < 0.01, ***p < 0.001 vs. NC; ^#^ *p* < 0.05, ^##^ *p* < 0.01, ^###^ *p* < 0.001 vs. DM; ^▲^ *p* < 0.01, ^▲▲^ *p* < 0.001 vs. Metformin. These statistical comparisons indicate significant differences between the groups in the study.

The findings indicated that serum HbA1c levels in the DM group were significantly elevated compared to the NC group (*p* < 0.001). After a 6-week treatment period, Metformin, Low dose, and High dose groups resulted in varying degrees of reduction in serum HbA1c levels compared to the DM group. The Metformin group showed a statistically significant decrease in HbA1c levels (*p* < 0.001), whereas both Low dose and High dose groups also exhibited significant reductions (*p* < 0.01). A statistically significant difference in HbA1c levels was noted between the Low dose group and the Metformin group (*p* < 0.05). The results are depicted in Figure 1A.

Following the 6-week treatment period, serum CP levels in the DM group were significantly lower than those in the NC group (*p* < 0.01). The CP levels in the Metformin group showed a significant increase compared to the DM group (*p* < 0.05), with the High dose group also demonstrating a significant rise (*p* < 0.05). However, no statistically significant differences (*p* > 0.05) were observed between the Metformin group and either the Low dose or High dose groups. The results are illustrated in Figure 1B.

### 3.4 Impact of CPCM on lipid metabolism in T2DM rats

#### 3.4.1 Impact of CPCM on triglycerides

Post-experimental analysis revealed that serum TG levels in DM group were significantly elevated compared to those in NC group (*p* < 0.001). After 6 weeks of intervention with metformin and CPCM, TG levels exhibited a significant decrease relative to the DM group (*p* < 0.001). However, no significant differences in TG levels were noted among the Metformin, Low dose, and High dose groups (*p* > 0.05). The results are depicted in Figure 1C.

#### 3.4.2 Impact of CPCM on total cholesterol

The data indicated that, after 6 weeks of continuous treatment, the DM group rats had significantly higher TC levels compared to the NC group (*p* < 0.001). In comparison to the DM group, TC levels in both the Metformin and High dose group showed significant reductions (*p* < 0.001), while the Low dose group also exhibited a significant decrease (*p* < 0.01). No significant differences in TC levels were observed among the treatment groups (*p* > 0.05). The results are illustrated in Figure 1D.

#### 3.4.3 Impact of CPCM on low-density lipoprotein cholesterol

In the DM group levels of LDL-C were significantly elevated compared to the NC group (*p* < 0.001). All treatment groups demonstrated a reduction in LDL-C levels relative to the DM group. Notable differences in LDL-C levels were present in both the Metformin and High dose groups when contrasted with the MD group (*p* < 0.001), while the Low dose group also exhibited a significant difference (*p* < 0.01). When compared to the Metformin group, the Low dose group showed statistically significant differences in LDL-C levels (*p* < 0.05). These findings are depicted in Figure 1E.

#### 3.4.4 Impact of CPCM on high-density lipoprotein cholesterol

Statistical analysis revealed no significant differences in HDL-C levels among the various groups (*p* > 0.05). The results are presented in Figure 1F.

### 3.5 Impact of CPCM on serum diamine oxidase, D-Lactic acid (D-Lactate), and bacterial endotoxin (LPS) levels in T2DM rats

#### 3.5.1 Impact of CPCM on serum diamine oxidase

The assessment of serum DAO levels in DM group indicated a significant increase compared to the NC group (*p* < 0.001). Additionally, serum DAO levels in the Metformin, High dose, and Low dose groups were significantly lower than those in the DM group (*p* < 0.001). Importantly, significant differences were noted when comparing the Low dose group to the Metformin group (*p* < 0.01). The results are illustrated in Figure 1G.

#### 3.5.2 Impact of CPCM on serum D-Lactic acid (D-Lactate)

Analysis of serum D-Lactate levels post-experimentation indicated that levels in DM group were significantly higher than in NC group (*p* < 0.001). In contrast to the DM group, both the Metformin and High dose, and Low-dose groups exhibited substantial decreases in D-Lactate levels, with extremely significant differences observed (*p* < 0.001). Furthermore, significant differences in D-lactate levels were identified when comparing the Low dose group to the Metformin group (*p* < 0.01). The results are presented in Figure 1H.

#### 3.5.3 Impact of CPCM on serum bacterial endotoxin

Data obtained after 6 weeks of continuous intervention showed that LPS levels in the DM group were significantly elevated compared to the NC group (*p* < 0.001). The Metformin, High dose, and Low dose groups demonstrated significant reductions in serum LPS levels compared to the DM group (*p* < 0.001). The Metformin, High-dose, and Low dose groups exhibited significant differences in LPS levels relative to the DM group (*p* < 0.001). Additionally, significant differences in LPS levels were found when comparing the Low dose group to the Metformin group (*p* < 0.01), and the High dose group also showed statistically significant differences (*p* < 0.05). The results are depicted in Figure 1I.

### 3.6 Impact of CPCM on serum inflammatory factors in T2DM rats

#### 3.6.1 Impact of CPCM on serum interleukin-1 beta

Post-experimental analysis revealed a significant elevation in serum IL-1β levels in DM rats compared to the NC group (*p* < 0.001). In the Metformin group, serum IL-1β levels demonstrated a significant reduction compared to the DM group (*p* < 0.001). Moreover, significant differences in IL-1β levels were detected between the High dose and Low-dose groups (*p* < 0.01). The Low dose group also exhibited significant differences in IL-1β levels in comparison to the Metformin group (*p* < 0.05). These findings are illustrated in Figure 1J.

#### 3.6.2 Impact of CPCM on serum tumor necrosis factor-alpha

After 6 weeks of sustained intervention, the data revealed that serum levels of TNF-α in the DM group were significantly elevated compared to those in the NC group (*p* < 0.001). In contrast, both the Metformin group and the High dose group exhibited a marked reduction in TNF-α levels when compared to the DM group, with statistically significant differences observed (*p* < 0.01). Furthermore, a significant difference in TNF-α levels was noted between the Low dose group and the Metformin group (*p* < 0.05). These results are illustrated in Figure 1K.

#### 3.6.3 Impact of CPCM on serum interleukin-6

Following 6 weeks of continuous treatment, the IL-6 levels in the DM group were significantly higher than those in the NC group (*p* < 0.001). Each intervention group demonstrated a decrease in IL-6 levels relative to the DM group, with statistically significant differences noted between the Metformin group and the High dose group (*p* < 0.05). However, no significant differences in IL-6 levels were observed among the Metformin, Low-dose, and High dose groups (*p* > 0.05). These findings are depicted in Figure 1L.

### 3.7 Impact of CPCM on mRNA expression levels of tight junction proteins and mucins in the colon tissues of T2DM rats

To investigate the effect of CPCM on the integrity of the colonic barrier, we quantified the mRNA expression levels of tight junction protein genes and mucin genes in the colon tissues of rats across different treatment groups. Quantitative PCR analysis indicated that, compared to the NC group, the mRNA expression levels of the tight junction proteins ZO-1, Occludin, and Claudin-1, as well as the mucin MUC2, were significantly downregulated in the colon of the DM group (*p* < 0.001). In the Metformin group, MUC2 expression levels were significantly elevated compared to the DM group (*p* < 0.001), alongside notable increases in ZO-1 and Occludin mRNA levels (*p* < 0.01). The increase in Claudin-1 levels was also statistically significant (*p* < 0.05). The High dose group exhibited a significant increase in ZO-1 expression compared to the DM group (*p* < 0.01). Moreover, the Low dose group displayed a significant enhancement in MUC2 expression (*p* < 0.001) and a statistically significant increase in Occludin levels (*p* < 0.05) compared to the DM group. When comparing the Low dose group to the Metformin group, significant differences in ZO-1 and Claudin-1 expression levels were observed (*p* < 0.05). The results of this study highlight the critical role of tight junction proteins and mucins in maintaining colonic barrier integrity, particularly in the context of compromised conditions, as seen in the DM group. The significant downregulation of these genes suggests that the colonic barrier may be severely impaired, which can lead to increased intestinal permeability and associated complications. The restoration observed in the Metformin and CPCM treatment groups suggests therapeutic potential for these agents in managing colonic barrier dysfunction. The fact that both treatments led to significant improvements in MUC2 and tight junction protein expressions points to their potential utility in clinical settings, particularly for conditions characterized by barrier dysfunction, such as inflammatory bowel diseases (IBD). Furthermore, the differential expression levels noted between the Low dose and Metformin groups suggest that the mechanisms of action may vary, warranting further investigation into the specific pathways through which CPCM and Metformin exert their effects. Understanding these mechanisms could lead to optimized treatment strategies that enhance colonic barrier function while minimizing potential side effects. Overall, this study presents compelling evidence for the protective effects of CPCM and Metformin on colonic barrier integrity, highlighting the importance of tight junction and mucin gene expression in maintaining a healthy intestinal environment. These results are presented in Figures 1M–P.

### 3.8 Impact of CPCM on mRNA expression levels and Western blot analysis of tight junction proteins (ZO-1, occludin, Claudin-1) and mucin (MUC2) in colon tissue of T2DM rats

Western blotting was conducted to evaluate the expression levels of tight junction proteins and mucins in the colon tissues of T2DM rats. The incorporation of β-actin as a loading control during the Western blot analysis is critical for ensuring the normalization of results and the reliability of experimental findings. The results revealed a significant decrease in the expression levels of tight junction proteins ZO-1, Claudin-1, and mucin MUC2 in the colon of rats in the DM group compared to the NC group (*p* < 0.001). Furthermore, Occludin expression was significantly reduced in the DM group (*p* < 0.01). In contrast, the Metformin group exhibited a marked increase in the levels of ZO-1, Claudin-1, and MUC2 proteins when compared to the DM group (*p* < 0.001), along with an enhancement in Occludin expression (*p* < 0.01). The Low dose group showed a significant upregulation of ZO-1 and MUC2 expression (*p* < 0.001), while Claudin-1 and Occludin also displayed notable increases (*p* < 0.01 and *p* < 0.05, respectively) in comparison to the DM group. Additionally, the High dose group presented a substantial increase in Claudin-1 and MUC2 expression relative to the DM group (*p* < 0.001), with a significant rise in Occludin expression (*p* < 0.05). Statistically significant differences in the expression levels of ZO-1 and Claudin-1 were observed between the Low dose group and the Metformin group (*p* < 0.05). Moreover, significant variation in Claudin-1 expression was recorded in the High dose group compared to the Metformin group (*p* < 0.05). The results of this study indicate that the expression of tight junction proteins and mucins in the colon tissue of T2DM rats is significantly suppressed, which may lead to compromised intestinal barrier function. This finding aligns with existing literature that highlights the detrimental effects of diabetes on gut barrier integrity, underscoring the importance of intestinal barrier maintenance for overall health. The significant therapeutic effects of Metformin are evident, as it effectively restores the expression of ZO-1, Claudin-1, and MUC2, potentially alleviating complications associated with T2DM through the enhancement of intestinal barrier function. Furthermore, CPCM demonstrates the ability to upregulate tight junction protein expression at both low and high doses, indicating its potential as a therapeutic agent, particularly in maintaining gut barrier integrity. It is noteworthy that differences in the expression of certain proteins between the low dose and high dose groups compared to the Metformin group suggest that varying treatment dosages may exert different biological effects. This provides important insights for future research aimed at exploring the mechanisms underlying CPCM and Metformin’s actions, as well as their implications for clinical applications. These findings are depicted in Figures 1Q–U.

### 3.9 Impact of CPCM on serum, hepatic, and fecal bile acids in T2DM rats

**FIGURE 2:**
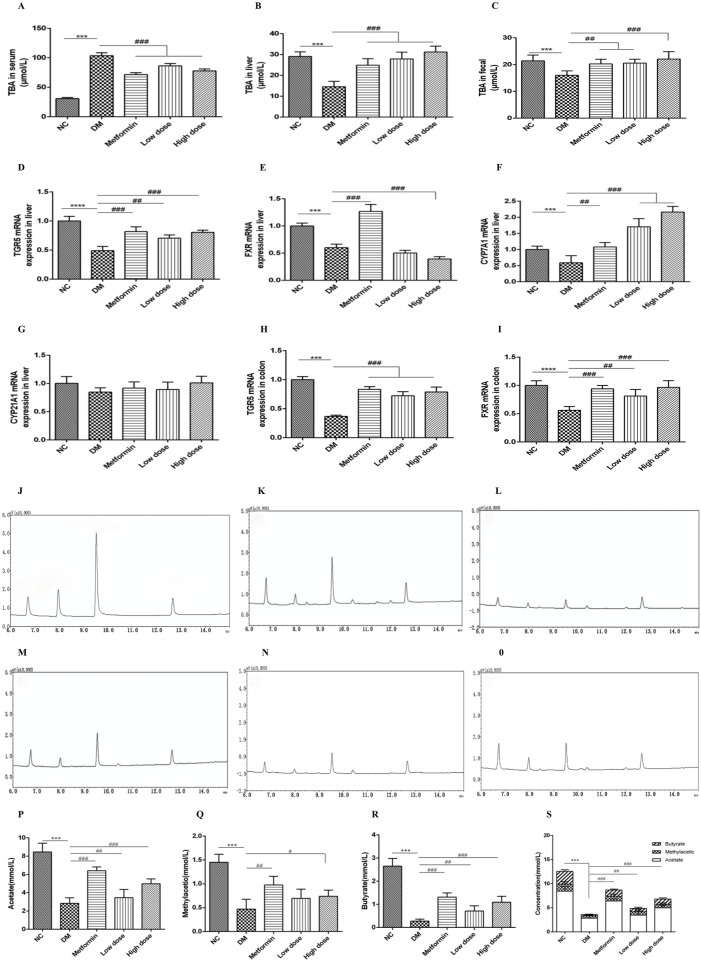
Impact of CPCM on bile acid metabolism and the expression of critical regulatory genes associated with bile acid homeostasis, along with gas chromatograms of short-chain fatty acids (SCFAs) from fecal samples, highlighting the concentrations of acetate, methyl acetate, butyrate, and total SCFAs in T2DM rats. **(A–C)** demonstrate the influence of CPCM on serum, hepatic, and fecal bile acid levels, respectively, in T2DM rats. Furthermore, **(D–G)** illustrate the effect of CPCM on hepatic mRNA expression of TGR5, FXR, CYP7A1, and CYP21A1 in the same cohort. **(H, I)** present the impact of CPCM on colonic mRNA expression of TGR5 and FXR, respectively. Gas chromatograms representing SCFAs from fecal samples in T2DM rats are shown, with **(J)** displaying the chromatogram of the standard sample. **(K–O)** depict gas chromatograms for the NC group, DM group, Metformin group, Low dose group, and High dose group, respectively. **(P–S)** illustrate the concentrations of acetate, methyl acetate, butyrate, and total SCFAs in fecal samples across different groups of T2DM rats. The study included a sample size of 8 rats, with results expressed as mean ± standard deviation (SD). ***p < 0.001 vs. NC; ^#^ *p* < 0.05, ^##^ *p* < 0.01, ^###^ *p* < 0.001 vs. DM. These statistical comparisons indicate significant differences between the groups in the study.

Following a 6-week treatment period, rats in the DM group demonstrated a marked elevation in serum total bile acid (TBA) concentrations relative to the NC group, while TBA levels in both liver and feces were significantly reduced (*p* < 0.001). In contrast to the DM group, the Metformin, High dose, and Low dose groups exhibited notable reductions in serum TBA levels, alongside significant increases in hepatic TBA levels (*p* < 0.001). Furthermore, both the Metformin and Low dose groups showed substantial increases in fecal TBA levels (*p* < 0.01), with the High dose group revealing a highly significant rise in fecal TBA levels (*p* < 0.001). These findings are illustrated in Figures 2A–C.

### 3.10 Impact of CPCM on hepatic mRNA expression of TGR5, FXR, CYP7A1, and CYP21A1 in T2DM rats

After 6 weeks of intervention, the DM group exhibited a significant downregulation of TGR5 mRNA expression in the liver compared to the NC group, with highly significant differences (*p* < 0.001). Relative to the DM group, both the Metformin and High dose groups demonstrated significant upregulation of TGR5 mRNA expression (*p* < 0.001), while the Low dose group also showed significant upregulation (*p* < 0.01).

Concerning FXR mRNA expression, the DM group displayed a significant downregulation compared to the NC group (*p* < 0.001). The Metformin group exhibited an upregulation of FXR mRNA expression compared to the DM group, with highly significant differences (*p* < 0.001). Conversely, the High dose group showed a further downregulation of hepatic FXR mRNA expression, which was statistically significant (*p* < 0.001), while no significant change was noted in the Low dose group (*p* > 0.05).

Regarding CYP7A1 mRNA expression, the DM group displayed a downregulation when compared to the NC group (*p* < 0.05). In contrast, the Metformin group demonstrated a significant upregulation of CYP7A1 mRNA expression (*p* < 0.01), with both High and Low dose groups exhibiting a highly significant upregulation (*p* < 0.001).

Lastly, there were no significant differences in CYP21A1 mRNA expression between the DM and NC groups (*p* > 0.05). Similarly, no significant differences were observed among the Metformin, High dose, and Low dose groups in relation to the DM group (*p* > 0.05). The results are depicted in Figures 2D–G.

### 3.11 Impact of CPCM on colonic mRNA expression of TGR5 and FXR in T2DM rats

After a 6-week intervention, the DM group rats exhibited a significant downregulation of TGR5 mRNA expression in the colon compared to the NC group, with highly significant differences (*p* < 0.001). In comparison to the DM group, the Metformin, High dose, and Low dose groups all showed significant upregulation of colonic TGR5 mRNA expression (*p* < 0.001).

With regard to FXR mRNA expression in the colon, the DM group displayed a significant downregulation relative to the NC group (*p* < 0.001). Conversely, both the Metformin and High dose groups exhibited significant upregulation of FXR mRNA expression when compared to the DM group (*p* < 0.001), while the Low dose group also showed significant upregulation (*p* < 0.01). These results are illustrated in Figures 2H, I.

### 3.12 Impact of CPCM on short-chain fatty acids in T2DM rats

#### 3.12.1 Analyte identification

Under the specified experimental conditions, three short-chain fatty acids (SCFAs) were successfully resolved. The chromatogram for the SCFA mixed standard is illustrated in Figure 2J. The retention times of the sample peaks matched those of the standards, thereby confirming the presence of acetate, methyl acetic, and butyrate with retention times of 6.73 min, 7.97 min, and 9.66 min, respectively.

#### 3.12.2 Linear regression analysis

Employing the internal standard approach within the chromatographic parameters established in this investigation, a standard curve was constructed with the peak area ratio plotted on the *y*-axis and concentration on the *x*-axis. The outcomes, detailed in Supplementary Table 1, reveal a robust linear correlation for acetate, methyl acetic, and butyrate.

#### 3.12.3 Method validation

To evaluate the method’s reliability, assessments were performed to analyze repeatability, stability, and accuracy. In the repeatability assessment, the identical fecal sample was analyzed in parallel six times, with the concentrations of each SCFA calculated. The relative standard deviation (RSD) values for acetate, methyl acetic, and butyrate were all below 3.42%, indicating excellent repeatability (refer to Supplementary Table 2).

In the stability evaluation, the same sample was analyzed at intervals of 0, 2, 4, 6, and 8 h. The RSD values for the concentrations of each SCFA over time ranged from 1.59% to 3.86%, confirming good sample stability (see Supplementary Table 3). Recovery experiments were conducted by spiking the sample with low, medium, and high concentrations of standard solutions, each analyzed in triplicate. The recovery percentages varied from 80.60% to 106.17%, thus demonstrating the method’s accuracy (see Supplementary Table 4).

#### 3.12.4 GC chromatograms of SCFAs in feces from various groups

The gas chromatography (GC) profiles indicated that the model group exhibited markedly reduced peak areas for acetate, methyl acetic, and butyrate in contrast to the normal group, with butyrate showing the most significant decline. Following treatment with metformin and high- and low-dose CPCM, the peak areas for acetate, methyl acetic, and butyrate were notably increased. The GC chromatograms for SCFAs in fecal samples from each group are presented in Figures 2K–O.

#### 3.12.5 Measurement of SCFA levels in feces from different groups

After 6 weeks of intervention, the fecal acetate levels in the DM group were significantly lower than those in the NC group (*p* < 0.001). Compared to the DM group, both the Metformin group and the High dose group displayed a significant elevation in acetate levels (*p* < 0.001), while the Low dose group did not show a statistically significant change (*p* > 0.05).

Similarly, after 6 weeks, the fecal methyl acetic levels in the DM model group were significantly diminished compared to the NC group (*p* < 0.001). The Metformin group demonstrated an increase in methyl acetic levels relative to the DM group (*p* < 0.01), and the High dose group also exhibited a statistically significant rise in methyl acetic (*p* < 0.05), whereas the Low dose group showed no significant variation (*p* > 0.05).

After 6 weeks of intervention, the fecal butyrate content in the DM group was significantly lower compared to the NC group (*p* < 0.001). Both the Metformin and High dose groups exhibited significant increases in butyrate levels compared to the DM group (*p* < 0.001), while the Low dose group showed a significant difference (*p* < 0.01).

Lastly, the total SCFA content in the feces of the DM group was significantly lower than that of the NC group (*p* < 0.001). In comparison to the DM group, both the Metformin and High dose groups demonstrated significant increases in total SCFA content (*p* < 0.001), while the Low dose group also showed a significant rise (*p* < 0.01). The findings are illustrated in Figures 2P–S.

### 3.13 Impact of CPCM on pancreatic, hepatic, and colon tissue architecture in T2DM rats

#### 3.13.1 Impact of CPCM on pancreatic tissue architecture in T2DM rats

**FIGURE 3:**
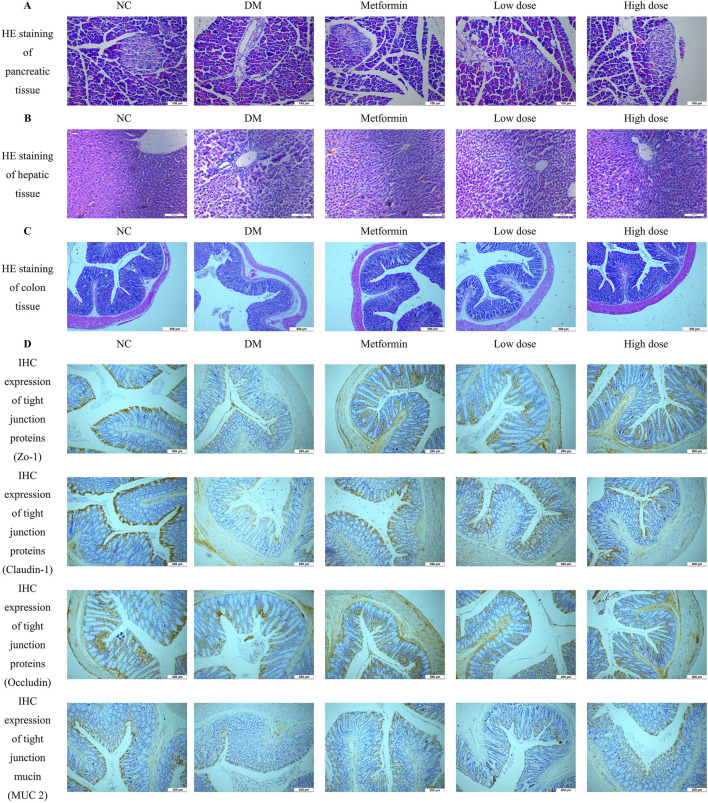
Impact of CPCM on Histological and Immunohistochemical Analysis of Pancreatic and Colon Tissue Architecture. **(A)** shows Hematoxylin and Eosin (HE) staining of pancreatic tissue architecture, with images captured at a magnification of ×200. The scale bar represents 100 µm. **(B)** presents HE staining of hepatic tissue architecture, also captured at a magnification of ×200, with a scale bar indicating 100 µm. **(C)** illustrates HE staining of colon tissue architecture, with images taken at a magnification of ×100 and a scale bar representing 500 µm. **(D)** displays the Immunohistochemistry (IHC) expression of tight junction proteins (ZO-1, Claudin-1, Occludin) and mucin (MUC2) in the colon of rats. Images were captured at a magnification of ×40, with a scale bar representing 200 µm. The analysis included a sample size of 8 rats per group.

Pancreatic tissue sections were prepared by fixation in 4% paraformaldehyde, followed by HE staining for histological examination. The pancreatic tissues from the NC group exhibited a high density of pancreatic islets, which appeared nearly elliptical, well-structured, and densely cellular. In contrast, the DM group demonstrated a marked reduction in the number of pancreatic islets, exhibiting irregular morphologies and signs of atrophy. The cellularity within the pancreatic islets was significantly diminished, resulting in discernible gaps in the tissue architecture. After treatment with Metformin and CPCM, both the Metformin and High dose groups displayed a reduction in vacuoles and gaps in the pancreatic tissue, coupled with an increase in the number of pancreatic islet cells. Similar enhancements were observed in the Low dose group. These findings are illustrated in Figure 3A.

#### 3.13.2 Impact of CPCM on hepatic tissue architecture in T2DM rats

Hepatic tissue sections were obtained through fixation in 4% paraformaldehyde, followed by HE staining for histopathological analysis. The liver tissues of the NC group rats displayed typical hepatocyte architecture, characterized by well-defined hepatic lobules and a regular arrangement of hepatic cords. Conversely, the DM group rats exhibited a disordered configuration of hepatic cords and altered hepatocyte morphology, accompanied by infiltration of inflammatory cells within the liver sinusoids. In comparison to the DM group, both the Metformin and CPCM-treated groups demonstrated a relatively organized structure of hepatic cords, with distinctly visible liver sinusoids and a marked reduction in pathological alterations. These findings are depicted in Figure 3B.

#### 3.13.3 Impact of CPCM on colon tissue structure in T2DM rats

Colon tissue samples were fixed in 4% paraformaldehyde for subsequent histopathological analysis. HE staining and morphological examination revealed that the colon tissue structure of the NC group was intact and well-organized, characterized by elongated and densely arranged intestinal villi. Conversely, the DM group exhibited an incomplete colonic architecture, characterized by damaged, shortened, and reduced intestinal villi. Additionally, the intestinal lumen was enlarged, resulting in irregular tissue organization. After a continuous 6-week intervention with Metformin and CPCM, the Metformin group showed a relatively preserved colonic structure, with improved organization and reduced damage to the intestinal villi, which appeared more densely arranged. The High dose group also demonstrated significant enhancements in colonic structural integrity, with increased length and density of the intestinal villi compared to the DM group. These results are depicted in Figure 3C.

### 3.14 Impact of CPCM on the expression levels of tight junction proteins and mucins proteins in the colon tissues of T2DM rats

IHC analysis revealed a significant downregulation of ZO-1, Claudin-1, Occludin, and MUC2 in the colon of the DM group compared to the NC group. Treatment with Metformin effectively mitigated the reduction of tight junction proteins in the colonic tissues of T2DM rats. Furthermore, both the high dose and low dose groups displayed comparable results, with expression levels of tight junction proteins closely resembling those found in the Metformin group. These findings suggest that CPCM has the potential to restore the expression levels of tight junction proteins and mucins in the colonic tissues of T2DM rats, as illustrated in Figure 3D.

## 4 Discussion

The increasing incidence of type 2 diabetes mellitus (T2DM) and its associated metabolic complications represent a substantial global health concern (Adler et al., 2024; El-Nashar et al., 2024a; El-Nashar et al., 2024b; ElSayed et al., 2024). In the current investigation, we evaluated the therapeutic efficacy of composite probiotics derived from fermented camel milk (CPCM) in a rat model of T2DM, focusing on metabolic dysregulation and intestinal barrier integrity.

### 4.1 CPCM dosage and effects

We prepared two distinct doses of the CPCM. The low-dose group received a formulation consisting of 1.0 × 10 ^ 8 CFU/mL of lactic acid bacteria and 1.0 × 10 ^ 6 CFU/mL of yeasts. In contrast, the high-dose group was administered a solution containing 1.0 × 10 ^ 10 CFU/mL of lactic acid bacteria and 1.0 × 10 ^ 8 CFU/mL of yeasts. Both formulations were dissolved in sterilized camel milk and administered at a dosage of 10 mL/kg. This method of preparation and administration was consistent across both groups (Manaer et al., 2021; Wang et al., 2020b). In this study, the high-dose treatment demonstrated a more pronounced effect compared to the low-dose treatment.

### 4.2 CPCM effects on T2DM rats

CPCM had a beneficial effect on the general condition of T2DM rats. This included increased activity levels, improved fur quality, and normalized feeding behaviors, suggesting a positive influence on the rats’ overall health and wellbeing. Furthermore, the observed improvements in body weight indicate enhanced metabolic regulation, highlighting its potential role in managing weight loss associated with diabetes. Importantly, a remarkable finding was the significant improvement in glycemic control. This was evidenced by a decrease in fasting blood glucose (FBG) levels by 6.5 mmol/L and a reduction in glycated hemoglobin (HbA1c) levels by 22.9%. Accompanied by enhanced insulin secretion as manifested by a 21.8% increase in C-peptide (CP) levels and a 40.1% reduction in the oral glucose tolerance test area under the curve (OGTT AUC). This strongly suggests that CPCM may directly or indirectly influence pancreatic β-cell function and insulin sensitivity. Each of these key indicators—fasting blood glucose (FBG), glycated hemoglobin (HbA1c), C-peptide (CP), and oral glucose tolerance test area under the curve (OGTT AUC)—plays a vital role in the management and understanding of diabetes. References for this statement include (Ojo, 2021; Ruszkiewicz et al., 2020; Livesey et al., 2019). The significant improvements observed in these parameters suggest that CPCM may be an effective intervention for enhancing glycemic control, promoting insulin secretion, and improving overall metabolic health in individuals with diabetes (Li et al., 2023). This aligns with previous research highlighting the beneficial effects of probiotics on glucose metabolism, although further investigation is needed to fully elucidate the underlying mechanisms (Manaer et al., 2015; Manaer et al., 2021; Wang et al., 2020b).

### 4.3 CPCM and gut microbiota

CPCM has a significant impact on gut microbiota composition and function. It leads to increased production of metabolites that interact with metabolic pathways involved in glucose homeostasis. Notably, the observed increases in fecal short-chain fatty acids (SCFAs), particularly butyrate (up by 289.9%), and a total SCFA elevation of 89.7%, may enhance insulin sensitivity and promote glucose uptake by peripheral tissues (Palmnäs-Bédard et al., 2022; Portincasa et al., 2022). This underscores the potential of CPCM as a therapeutic strategy for improving metabolic health, as elevated SCFAs are linked to better insulin sensitivity and glucose management—critical factors in diabetes management and complication prevention (Xiong et al., 2022). These findings suggest that targeting gut microbiota through interventions like CPCM could represent a novel approach to support glucose homeostasis and overall metabolic function.

### 4.4 CPCM and lipid profiles

The observed improvements in lipid profiles from CPCM treatment are particularly advantageous for individuals with T2DM. Given the prevalence of dyslipidemia in this population and its strong correlation with cardiovascular disease risk. A significant reduction in total cholesterol (TC) by 40.0% is crucial, as elevated TC levels are a major indicator of atherosclerotic risk, contributing to plaque formation in arteries (DiNicolantonio et al., 2016). This reduction can lower the risk of cardiovascular events, which is vital for T2DM patients who are already predisposed to heart disease (Khil et al., 2023). Additionally, the reduction in triglycerides (TG) by 17.1% is noteworthy since high TG levels are common in diabetes and are linked to insulin resistance and cardiovascular issues (Budoff, 2016). Lowering TG can enhance overall metabolic health and decrease the risk of complications such as pancreatitis associated with hypertriglyceridemia (Callaghan et al., 2011). Furthermore, the reduction in low-density lipoprotein cholesterol (LDL-C) by 30.4% is particularly beneficial, as LDL-C is known as “bad cholesterol” for its role in fatty deposit accumulation in arteries (Katzmann and Laufs, 2019). A significant decrease in LDL-C can mitigate the risk of cardiovascular diseases and improve overall heart health (He et al., 2024). The lipid-lowering effects of CPCM likely involve multiple mechanisms. For example, short-chain fatty acids (SCFAs) play a role. SCFAs can suppress hepatic lipogenesis, which is the process of fat production in the liver. By suppressing this process, it leads to reduced fat production and promotes cholesterol excretion, thereby lowering circulating cholesterol levels (Li et al., 2022). Additionally, CPCM may modulate gene expression related to lipid metabolism, such as the upregulation of TGR5 and CYP7A1 in the liver, which are involved in bile acid synthesis. This indicates that CPCM enhances the liver’s ability to manage lipids effectively (Chiang and Ferrell, 2022; Yang et al., 2023). Upregulation of TGR5 and FXR in the colon also suggests that CPCM may positively influence gut-liver axis interactions, which are vital for maintaining lipid homeostasis (Chen and Cassaro, 2023). Overall, the marked reductions in TC, TG, and LDL-C following CPCM treatment are highly relevant for managing dyslipidemia in T2DM patients, as they not only promote metabolic health but also significantly reduce the risk of cardiovascular complications, a leading cause of morbidity and mortality in individuals with diabetes.

### 4.5 CPCM and biomarkers

CPCM’s effects on serum diamine oxidase (DAO), D-lactic acid (D-lactate), and bacterial endotoxin (LPS) levels in T2DM rats reveal important connections between these biomarkers and diabetes pathology. Elevated DAO levels indicate compromised intestinal barrier function, leading to increased gut permeability and systemic inflammation, which exacerbates insulin resistance and metabolic dysfunction (Imai et al., 2024). The significant reduction in DAO levels post-CPCM treatment suggests enhanced gut integrity. Elevated D-lactate levels, often resulting from gut bacterial overgrowth, indicate impaired gut health and are linked to metabolic disorders (Remund et al., 2023). CPCM’s ability to substantially lower D-lactate suggests a restoration of gut microbial balance, promoting metabolic health. Additionally, increased LPS levels signal systemic inflammation associated with insulin resistance and diabetes progression (Vatanen et al., 2016). The significant decrease in LPS levels following CPCM treatment indicates potential mitigation of inflammatory responses, reducing the risk of diabetes-related complications. Overall, the reduction of DAO, D-lactate, and LPS levels after CPCM treatment highlights its potential to improve gut health and reduce inflammation in T2DM. These effects not only address metabolic dysfunction but also help prevent long-term complications, enhancing patients’ overall health and quality of life.

### 4.6 CPCM and inflammatory markers

The modulation of inflammatory markers like interleukin-1β (IL-1β), tumor necrosis factor-α (TNF-α), and interleukin-6 (IL-6) is crucial in T2DM, as their elevated levels are associated with disease progression, insulin resistance, and β-cell dysfunction. IL-1β promotes inflammation and impairs insulin signaling, worsening hyperglycemia (Chen et al., 2021). TNF-α interferes with insulin pathways, fostering a state of chronic inflammation that can lead to complications such as cardiovascular disease (Akash et al., 2018). Similarly, high IL-6 levels are associated with insulin resistance and disrupt glucose metabolism (Qu et al., 2014). The significant reduction of IL-1β, TNF-α, and IL-6 after CPCM treatment highlights its anti-inflammatory properties. By lowering these cytokines, CPCM may improve insulin sensitivity and metabolic control in T2DM. Overall, the relationship between these inflammatory markers and T2DM is critical, and CPCM’s ability to modulate their levels suggests a potential therapeutic role in managing diabetes-related inflammation and improving patient outcomes.

### 4.7 CPCM and bile acids

In the high-dose CPCM group, there were notable reductions in serum total bile acid (TBA) levels, while significant increases were observed in both hepatic and fecal TBA levels. These changes suggest improved metabolic regulation, which has important implications for diabetes management. Enhanced bile acid dynamics may contribute to better glycemic control, as bile acids are known to play a role in glucose metabolism and insulin sensitivity (Feng et al., 2021). By promoting the elimination of bile acids and improving their regulation, CPCM may help mitigate the metabolic disturbances associated with T2DM, potentially leading to better overall health outcomes for individuals with the condition.

### 4.8 CPCM and tissue improvements

High-dose CPCM treatment has demonstrated remarkable effects on pancreatic, hepatic, and colonic tissues, promoting significant structural improvements and the recovery of vital cellular functions. One of the key outcomes observed was the enhancement of pancreatic tissue architecture, characterized by a notable increase in the number of pancreatic islet cells and a reduction in vacuoles and gaps within the tissue. This restoration is crucial for improving insulin secretion capabilities, which is vital for better blood glucose regulation in individuals with T2DM (Adams and Blum, 2022). Moreover, the colonic tissue from subjects receiving high-dose CPCM showed significant enhancements, including increased length and density of intestinal villi and reduced tissue damage. Such improvements in colonic structure are essential for maintaining gut health and function, promoting better nutrient absorption and overall digestive wellness, particularly important for diabetic patients who often experience gastrointestinal issues (Eslami et al., 2021).

Furthermore, high-dose CPCM treatment was found to restore the expression of tight junction proteins, such as ZO-1, Claudin-1, and Occludin, as well as mucin (MUC2) in colonic tissues. The recovery of these proteins is vital for maintaining the integrity of the intestinal barrier, which can prevent the translocation of harmful substances and bacteria, thereby reducing the risk of inflammation and other gastrointestinal complications associated with diabetes. ZO-1 (Zonula Occludens-1) is a vital scaffolding protein at tight junctions between epithelial cells, essential for maintaining the integrity and stability of the epithelial barrier (Itoh et al., 2018). By connecting transmembrane proteins to the actin cytoskeleton, ZO-1 regulates cell polarity and intercellular communication, and its reduced expression can compromise barrier function, permitting harmful substances to penetrate the intestinal lining (Kuo et al., 2021). Claudin-1 is a crucial component of tight junctions that enhances the sealing properties of the epithelial barrier and regulates the permeability of the intestinal lining, controlling the passage of ions and small molecules (Fernández-García et al., 2024). Adequate levels of Claudin-1 are essential for preventing the leakage of pathogens and toxins into the bloodstream, and its disruption is linked to various gastrointestinal disorders and increased intestinal permeability (Safari et al., 2023), commonly known as “leaky gut.” Occludin is an integral membrane protein in tight junctions that plays a crucial role in maintaining barrier function and regulating cell-to-cell adhesion (Torices et al., 2023). Occludin interacts with ZO-1 and Claudin-1 to strengthen tight junction structure, and its altered expression has been associated with inflammatory conditions and compromised intestinal barrier function, highlighting its importance in preventing the translocation of harmful bacteria and substances (Duan et al., 2021). MUC2 is a gel-forming mucin primarily secreted by goblet cells in the intestinal epithelium, and it is essential for forming a protective mucus layer that serves as a barrier against pathogens and helps maintain a healthy microbiome (Yao et al., 2021). MUC2 also provides lubrication and protects epithelial cells from mechanical and chemical damage, and adequate expression of MUC2 is critical for gut health, as its deficiency can increase susceptibility to infections and inflammation (Yamashita and Melo, 2018).

Although this study demonstrated the beneficial effects of CPCM on T2DM rats, there are some limitations. For example, the study was conducted in a rat model, and the results may not directly translate to humans. Future studies are needed to investigate the efficacy and safety of CPCM in human subjects with T2DM.

## 5 Conclusion

The use of CPCM demonstrated numerous beneficial effects in rats with T2DM. This study evaluated the therapeutic efficacy of composite probiotics derived from fermented camel milk (CPCM) in a rat model of type 2 diabetes mellitus (T2DM). The findings suggest that CPCM has several beneficial effects on T2DM rats. CPCM at different doses had varying impacts, with the high-dose treatment showing more pronounced effects. It improved the general condition of T2DM rats, including increased activity levels, better fur quality, and normalized feeding behaviors. Additionally, it enhanced metabolic regulation as indicated by improvements in body weight and significant improvements in glycemic control, manifested by decreased fasting blood glucose and glycated hemoglobin levels, increased C-peptide levels, and reduced oral glucose tolerance test area under the curve. This suggests a potential role for CPCM in influencing pancreatic β-cell function and insulin sensitivity. CPCM also had a significant impact on gut microbiota composition and function. It increased the production of metabolites that interact with metabolic pathways involved in glucose homeostasis, particularly short-chain fatty acids. Elevated levels of these fatty acids may enhance insulin sensitivity and promote glucose uptake by peripheral tissues. Furthermore, CPCM treatment led to improvements in lipid profiles, reducing total cholesterol, triglycerides, and low-density lipoprotein cholesterol. These reductions are crucial for managing dyslipidemia in T2DM patients and reducing the risk of cardiovascular complications. The lipid-lowering effects likely involve multiple mechanisms, including the role of short-chain fatty acids and modulation of gene expression related to lipid metabolism. The effects of CPCM on serum biomarkers such as diamine oxidase, D-lactic acid, and bacterial endotoxin levels highlight its potential to improve gut health and reduce inflammation in T2DM. Additionally, CPCM modulated inflammatory markers like interleukin-1β, tumor necrosis factor-α, and interleukin-6, suggesting an anti-inflammatory role and potential for improving insulin sensitivity and metabolic control. In the high-dose CPCM group, there were notable changes in serum total bile acid levels, along with improvements in pancreatic, hepatic, and colonic tissues. High-dose CPCM promoted structural improvements in these tissues and restored the expression of tight junction proteins and mucin in colonic tissues, enhancing intestinal barrier integrity. However, this study has some limitations. As it was conducted in a rat model, the results may not directly translate to humans. Future studies are needed to investigate the efficacy and safety of CPCM in human subjects with T2DM. Overall, CPCM shows promise as a potential therapeutic intervention for T2DM, but further research is required to fully understand its mechanisms and applicability in humans.

## Data Availability

The raw data supporting the conclusions of this article will be made available by the authors, without undue reservation. Requests to access these datasets should be directed to XN, xh2022@xjmu.edu.cn.

## Glossary

AUC: Area Under Curve
BW: Body Weight
CP: C-peptide
CPCM: Composite probiotics derived from fermented camel milk
CFU: Colony-Forming Units
DAO: diamine oxidase
DM: Diabetes Mellitus
DM: diabetes model group
D-Lactate: D-Lactic Acid
ELISA: Enzyme-Linked Immunosorbent Assay
FBG: Fasting Blood-Glucose
FID: flame ionization detector
GC: Gas Chromatography
GLUT4: Glucose Transporter 4
HE: Hematoxylin and Eosin
HbA1c: Glycosylated Hemoglobin
HDL-C: High-Density Lipoprotein;
High dose: high-dose group
IACUC: Institutional Animal Care and Use Committee
IBD: inflammatory bowel diseases
IHC: Immunohistochemistry
IL-1β: Interleukin-1 Beta
IL-6: Interleukin-6
IL-8: Interleukin-8
IRI: Insulin Resistance Index
ISAPP: International Scientific Association for Probiotics and Prebiotics
LAB: lactic acid bacteria
LBP: Lipopolysaccharide-Binding Protein
LDL-C: Low-Density Lipoprotein
Low dose: low-dose group
LPS: Lipopolysaccharide
Metformin: Metformin group
mRNA: Messenger Ribonucleic Acid
MRS: De Man, Rogosa, and Sharpe
NC: normal control group
ANOVA: one-way analysis of variance
OGTT: Oral Glucose Tolerance Test
PCR: Polymerase Chain Reaction
RSD: relative standard deviation
SCFAs: Fecal short-chain fatty acids
SD: standard deviation
SPF: specific pathogen-free
STZ: streptozotocin
TBA: total bile acid
T2DM: Type 2 diabetes
TC: Total Cholesterol
TG: Triglyceride
TLRs: Toll-Like Receptors
TNF-α: Tumor Necrosis Factor-alpha
ZO-1: Zonula Occludens-1
