# Supplementary material for: Therapeutic effects of composite probiotics derived from fermented camel milk on metabolic dysregulation and intestinal barrier integrity in type 2 diabetes rats
Source: Front Pharmacol. 2025 Jan 7;15:1520158. doi: 10.3389/fphar.2024.1520158 (PMC11747018; doi:10.3389/fphar.2024.1520158)
Supplement: Supplementary file 1 [file Table1.docx]

Supplementary Table 1. Standard Equations and Correlation Coefficients of SCFAs

| Name | Regression Equation | Correlation Coefficient (R) | Linear Range (mmol/L) |
| --- | --- | --- | --- |
| Acetate | Y = 0.1592X + 0.0143 | 0.9995 | 0.188-12.010 |
| Methylacetic | Y = 0.3667X + 0.0820 | 0.9993 | 0.116-7.400 |
| Butyrate | Y = 1.0612X + 0.0851 | 0.9996 | 0.138-8.811 |

Supplementary Table 2. Repeatability Measurement Results of SCFAs

| n | 1 | 2 | 3 | 4 | 5 | 6 | RSD(%) |
| --- | --- | --- | --- | --- | --- | --- | --- |
| Acetate(mmol/L) | 7.46 | 7.40 | 7.69 | 7.54 | 7.59 | 7.42 | 1.48 |
| Methylacetic(mmol/L) | 1.41 | 1.49 | 1.43 | 1.47 | 1.38 | 1.37 | 3.42 |
| Butyrate(mmol/L) | 2.61 | 2.76 | 2.64 | 2.55 | 2.73 | 2.76 | 3.26 |

Supplementary Table 3. Stability Measurement Results of SCFAs

| Time(h) | 0 | 2 | 4 | 6 | 8 | RSD（%） |
| --- | --- | --- | --- | --- | --- | --- |
| Acetate(mmol/L) | 6.88 | 6.83 | 6.84 | 6.74 | 6.68 | 1.59 |
| Methylacetic(mmol/L) | 1.03 | 1.02 | 0.99 | 0.99 | 0.96 | 3.86 |
| Butyrate(mmol/L) | 2.66 | 2.64 | 2.65 | 2.62 | 2.56 | 1.99 |

Supplementary Table 4 Recovery results of SCFAs

| SCFA Name | Baseline Concentration (mmol/L) | Spiked Concentration (mmol/L) | Measured Concentration (mmol/L) | Recovery (%) | Mean Recovery (%) |
| --- | --- | --- | --- | --- | --- |
| Acetate | 4.21±0.27 | 3 | 6.92±0.41 | 93.17 | 93.06 |
|  | 4.21±0.27 | 4 | 7.58±0.56 | 84.79 |  |
|  | 4.21±0.27 | 5 | 9.25±0.18 | 101.21 |  |
| Methylacetic | 1.50±0.09 | 1 | 2.21±0.14 | 80.61 | 94.12 |
|  | 1.50±0.09 | 2 | 2.43±0.17 | 95.59 |  |
|  | 1.50±0.09 | 3 | 4.59±0.04 | 106.17 |  |
| Butyrate | 2.77±0.14 | 2 | 4.41±0.21 | 87.09 | 87.06 |
|  | 2.77±0.14 | 3 | 4.54±0.27 | 91.18 |  |
|  | 2.77±0.14 | 4 | 6.31±0.21 | 82.90 |  |
